# Flower-Like CuO/ZnO Hybrid Hierarchical Nanostructures Grown on Copper Substrate: Glycothermal Synthesis, Characterization, Hydrophobic and Anticorrosion Properties

**DOI:** 10.3390/ma10070697

**Published:** 2017-06-25

**Authors:** Farshad Beshkar, Hossein Khojasteh, Masoud Salavati-Niasari

**Affiliations:** Institute of Nano Science and Nano Technology, University of Kashan, Kashan 87317-51167, Iran; f.beshkar69@yahoo.com (F.B.); hn.khojasteh@gmail.com (H.K.)

**Keywords:** CuO film, flower-like ZnO, hydrophobicity, contact angle, anticorrosion

## Abstract

In this work we have demonstrated a facile formation of CuO nanostructures on copper substrates by the oxidation of copper foil in ethylene glycol (EG) at 80 °C. On immersing a prepared CuO film into a solution containing 0.1 g Zn(acac)_2_ in 20 mL EG for 8 h, ZnO flower-like microstructures composed of hierarchical three-dimensional (3D) aggregated nanoparticles and spherical architectures were spontaneously formed at 100 °C. The as-synthesized thin films and 3D microstructures were characterized using XRD, SEM, and EDS techniques. The effects of sodium dodecyl sulphate (SDS), cetyltrimethylammonium bromide (CTAB), and polyethylene glycol (PEG) 6000 as surfactants and stabilizers on the morphology of the CuO and ZnO structures were discussed. Possible growth mechanisms for the controlled organization of primary building units into CuO nanostructures and 3D flower-like ZnO architectures were proposed. The hydrophobic property of the products was characterized by means of water contact angle measurement. After simple surface modification with stearic acid and PDMS, the resulting films showed hydrophobic and even superhydrophobic characteristics due to their special surface energy and nano-microstructure morphology. Importantly, stable superhydrophobicity with a contact angle of 153.5° was successfully observed for CuO-ZnO microflowers after modification with PDMS. The electrochemical impedance measurements proved that the anticorrosion efficiency for the CuO/ZnO/PDMS sample was about 99%.

## 1. Introduction

Copper is widely applied in many applications such as the electronic industries and communications as a conductor in electrical power lines, pipelines for domestic and industrial water utilities including seawater, heat conductors, and heat exchangers [1]. Due to their special physical properties and high potential for various electronic and photonic device applications, semiconductors have attracted wide attention [2]. CuO is a p-type semiconductor that has a small band gap (Eg = 1.2 eV) and it is one of the most important and versatile semiconductors that is widely used as superconductors, catalysts, a potential field-emission material, and that has high-temperature durability [3,4]. Due to these properties, copper and its alloys have been one of the important materials in industry. Thus, the protection of used copper has particular importance in the industry. One of the effective methods for surface protection of metals against external threats is by making their surface hydrophobic to repulse water and its solved corrosion containing materials. 

On the other hand, zinc oxide (ZnO) is a famous n-type semiconductor (Eg = 3.37 eV at 300 K) that has a high electron mobility and low recombination loss [5]. Because of its semiconducting, piezoelectric, and pyroelectric attributes, ZnO has become one of the most important semiconductor materials used for diverse applications such as optoelectronics, gas sensing, catalysis, solar cell, and actuators [6,7,8,9].

Producing hybrid nanostructured materials in order to achieve various properties such as high surface area, good conductivity, and permeability, have been considered to be one of the most important functional materials for the various research fields [10,11]. Some investigations have been reported for directly constructing ZnO nano or micro-structures on copper substrates. The combination of CuO and ZnO nanostructures has been reported, but at high temperature to oxidize a copper foil to CuO followed by the deposition of ZnO on the copper oxide surface [12]. As a result, many CuO/ZnO hybrid nanostructures have been fabricated so far, including CuO/ZnO core/shell heterostructures produced by thermal decomposition [13], CuO/ZnO nanocomposites for non-enzymatic glucose sensing applications [14], ZnO/CuO hetero-hierarchical nanotrees prepared by hydrothermal preparation [15], and flower-like CuO-ZnO structured nanowires by chemical deposition [16].

In addition, it has been reported that the hierarchical nanorod-like structure of the CuO/ZnO photoelectrode provides higher surface area, more reactive sites, effective light absorption, and reduced charge transfer resistance at the electrode/electrolyte interface that showed superior photoelectrochemical properties. Also, the photo conversion efficiency and stability of the CuO/ZnO photoelectrode were enhanced due to the formation of p-n junctions along the p-CuO core and n-ZnO protective shell, respectively [17].

As we know the performance of hybrid nanomaterials depends upon their size, morphology, composition, dispersion, structure, crystal phases, and crystal facets [18]. The surface of a material is the most important factor determining the compatibility and type of interaction with its environment [19]. In addition, the wetting behavior is one of the most important characteristic of a solid surface and is strongly influenced by the size and morphology of the surface particle structure. This property has a particular effect on the life span, energy consumption, and practical purposes of engineered materials [20]. Therefore, the controlled synthesis of hybrid metal oxide nanostructures is of prominent interest to obtain the required grain size, surface morphology, and structure of the hybrid nanostructure.

In the present work, flower-like ZnO micro/nanostructures fabricated on a copper substrate were produced by a facile, affordable, and environmentally-friendly glycothermal method in the presence of ethylene glycol as reduction agent and green solvent. Meanwhile, the effects of different surfactants on the final surface morphology and coarseness were investigated. The wetting and anticorrosion properties of the samples were also studied by the measurement of the contact angle and electrochemical impedance, respectively.

## 2. Experimental Section

### 2.1. Materials and Methods

Copper foils, hydrochloric acid (37%), glacial acetic acid, ethylene glycol (EG), cetyltrimethylammonium bromide (CTAB), sodium dodecyl sulphate (SDS), polyethylene glycol 6000 (PEG 6000), and zinc acetylacetonate (Zn(acac)_2_) were purchased from Merck Company. Polydimethylsiloxane (PDMS) was provided by Sigma-Aldrich. Ammonia solution (25%), stearic acid, and ethanol were kindly provided by Ghatran Shimi Co (Tehran, Iran). All materials and solvents were used without further purification. Deionized water (DI water) was used throughout. GC-2550TG (Teif Gostar Faraz Company, Tehran, Iran) were used for all chemical analyses. The synthesized architecture materials were characterized using various analytical methods. XRD patterns were recorded by a Philips, X-ray diffractometer (Philips, Egham, England) using Ni-filtered Cu Kα radiation. The morphology of the products was measured using a Hitachi S-4160 field emission scanning electron microscope (FESEM, Hitachi, Ltd., Tokyo, Japan). Prior to taking images, the samples were coated with a very thin layer of Pt to make the sample surface conducting and prevent charge accumulation. The energy dispersive spectrometry (EDS) analysis was conducted using a Tescan mira3 microscope (Hitachi, Ltd., Tokyo, Japan). Contact angles (CAs) were measured using a contact-angle meter (Dataphysics, OCA 15 plus, DataPhysics Instruments GmbH, Filderstadt, Germany) equipped with a CCD camera at room temperature. The volume of water drip was approximately 4 µL. The average CA value was reported by measuring the right and left positions for the droplet.

### 2.2. Synthesis of Nanoscale CuO Arrays on the Cu Substrate

**Figure 1:**
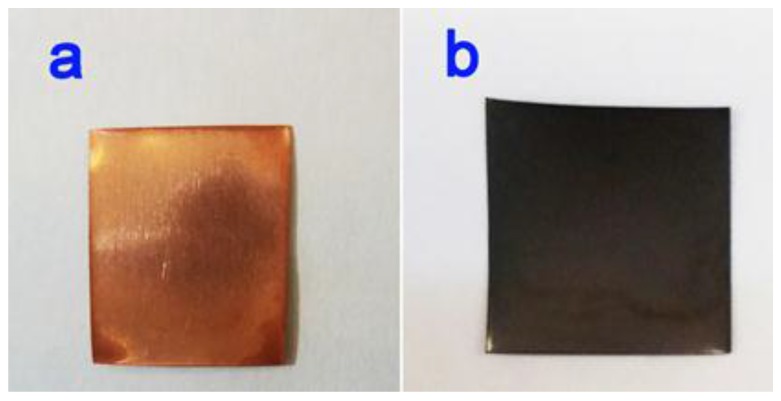
The appearance of the Cu foils between the different stages treatments. (**a**) the cleaned Cu foil; (**b**) the Cu foil reacted with EG at 80 °C for 5 days.

The CuO nanoscale array was prepared via in situ engraving of the Cu foil (1 cm × 1 cm) in alkaline conditions. Briefly, the Cu foils were immersed in 10 mL of 3 M HCl aqueous solution and ultrasonicated for about 10 min to refresh the surface and remove the surface impurities and oxide layers. After that, the foils were rinsed with ethanol and distilled water two times for 10 min in a sonication bath, respectively, and then were dried at 60 °C for 1 h in air atmosphere (Figure 1a). At the second step, the cleaned Cu foils were immersed into a sealed beaker containing 2.8 mL of concentrated HCl, 1.7 mL of acetic acid, and 10 mL of deionized water solution, and the reaction was performed at room temperature for 6 h. In this step, a rough surface for the modification and growth of the other material will be achieved. The change in the foil appearance during the reaction is shown in Figure 1b. As seen in the Figure 1a, the surface of the copper foil became bright and smooth after the cleaning treatment.

The prepared Cu foils were placed in 10 mL of EG at 80 °C for 5 days. The obtained samples were rinsed in DI water and then dried in air at 60 °C for 30 min. After drying, the uniformly black surface layer of the CuO nanosphere array growing on the Cu foil was obtained (Figure 1b). Then the surface morphology of CuO in the absence (C0 sample) or presence of 0.2 g of different stabilizers or surfactants (C1 = CTAB, C2 = SDS, and C3 = PEG6000) were investigated. The detailed preparation conditions are summarized in Table 1.

### 2.3. Preparation of 3D CuO-ZnO Hybrid Hierarchical Structures

**Scheme 1:**
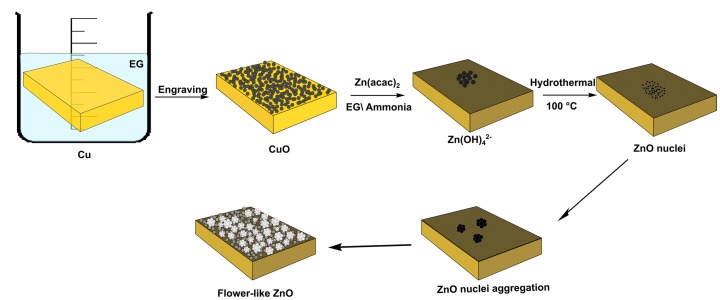
Schematic illustration of the flower-like CuO-ZnO synthesis procedure.

To prepare the CuO-ZnO flower-like structures, a simple glycothermal method was used. A schematic illustration of the synthesis of flower-like ZnO is shown in Scheme 1. A solution containing 0.1 g of Zn(acac)_2_ in 20 mL EG was prepared and stirred for several minutes to obtain a homogeneous solution. The needed amount of ammonia was added dropwise to the solution and the pH was adjusted to 10. After that, the previously prepared foils were immersed in the obtained solution. The prepared mixture was transferred to a 250 mL Teflon-lined autoclave and the substrate was hung in the Teflon container. The reaction was performed in glycothermal conditions at 100 °C for 8 h. Finally, the samples were rinsed in ethanol and DI water three times and then dried in air at 60 °C for 6 h. Here, the effects of the absence (Z0 sample) or presence of different stabilizer or surfactants (Z1 = CTAB, Z2 = SDS, and Z3 = PEG6000) with specific molar ratios (Zn:Surfactant = 1:2) on the final produced structures were investigated. The detailed preparation conditions are mentioned previously in Table 1. 

### 2.4. Anticorrosion Behaviour of the Superhydrophobic CuO-ZnO Film

Electrochemical studies were carried out using an AUTOLAB model PGSTAT 30. To investigate the anticorrosion ability, Tafel plots and electrochemical impedance spectroscopy (EIS) tests were used by a conventional three-electrode cell (the platinum foil as the counter electrode, Ag/AgCl/KCl (3 mol·L^−1^) as the reference electrode, and the foil with an exposed area of 1 cm^2^ as the working electrode) with a capacity of 100 mL. All impedance curves were recorded at room temperature (25 ± 2 °C). The working electrode was immersed in the test solution (in aqueous NaCl solution (3.5%)) for 60 min until a steady state open-circuit potential was attained. The EIS measurements were performed over the frequency range from 100 kHz to 100 mHz at the open circuit potential by superimposing the alternating current (AC) signal of 0.01 V after immersion for 60 min in the corrosive media. NOVA software was used for fitting the impedance data in an equivalent circuit as well as for extrapolating the Tafel slopes. Experiments were carried out in triplicate to ensure the reproducibility of the results. The inhibition efficiencies (η) for each inhibitor concentration were calculated using Equation (1):

(1) $$\eta \%=\frac{R_{\mathrm{Ct}}-R_{0}}{R_{\mathrm{Ct}}}\times 100$$

where R_ct_ and R_0_ are the charge transfer resistances in the presence and absence of the inhibitor, respectively.

## 3. Results and Discussion

X-ray powder diffraction (XRD) is a rapid, accurate, and nondestructive technique used for chemical and physical analysis of the materials. It is mostly used for phase identification of a crystalline material and can provide information on the unit cell dimensions. To compare and confirm the phase composition of the product, here the XRD experiment was carried out.

**Figure 2:**
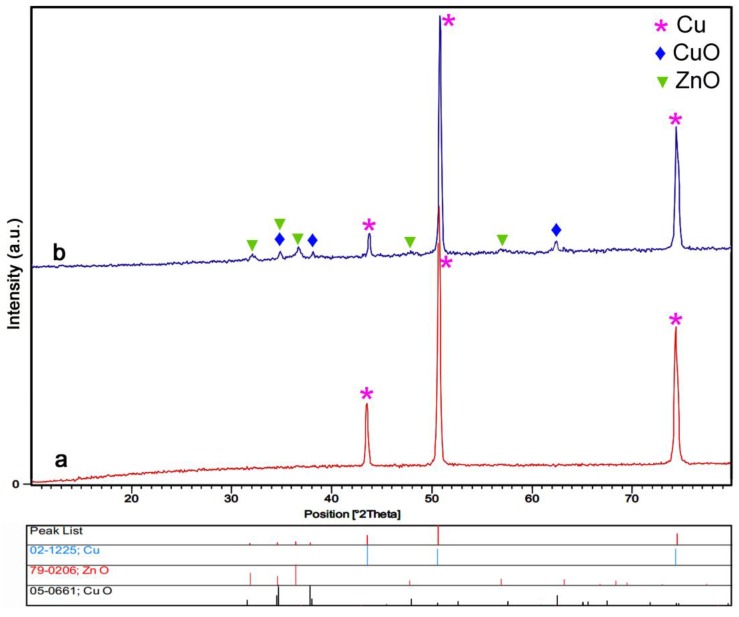
Wide-angle XRD patterns of the clean Cu foil obtained after cleaning treatment stage (**a**) and the as-prepared flower-like CuO-ZnO micro/nanostructures obtained after the engraving and glycothermal stages (**b**).

For evaluation of the crystal structure and purity of the as-prepared samples, wide-angle XRD patterns of clean Cu foil (C0 sample) and the as prepared final product (Z0 sample) were taken. The results are shown in Figure 2 as a comparison. As seen in Figure 2a, the Cu diffraction peaks are very strong and all the peaks corresponding to the face-centered cubic Cu were well matched with the database in JCPDS (File No. 02-1225).

After interaction with acid and Zn(acac)_2_, the XRD pattern of the product grown on the upward surface of the copper foil demonstrates the presence of phases, a monoclinic structure for CuO, and a hexagonal structure for flower-like ZnO (Figure 2b). It could be seen that the ZnO and CuO diffraction peaks are very weak, which is likely due to the small amount formed on the clean Cu substrate. The observed lattice constant values are in good agreement with the standard values with JCPDS file no. 79-0206 (a = 3.249 Å, c = 5.206 Å) for ZnO and JCPDS file no 05-066 (a = 4.688 Å, b = 3.423 Å, c = 5.132 Å) for the CuO crystals. The XRD pattern confirmed that the synthesis was successful.

**Figure 3:**
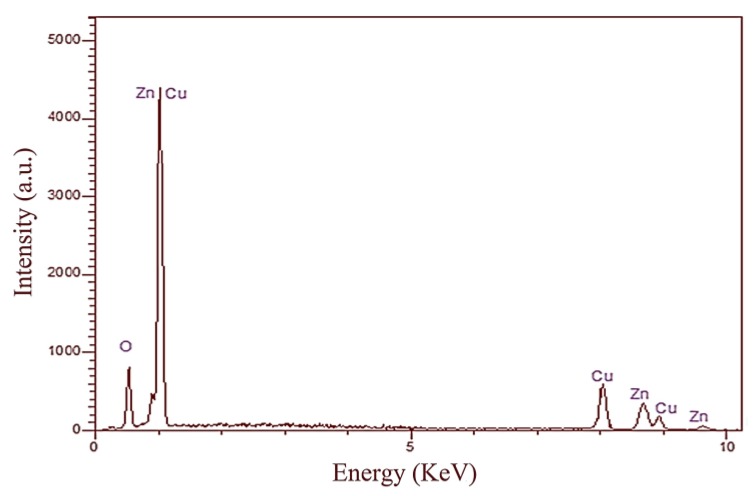
EDS spectrum of the flower-like CuO-ZnO hybrid hierarchical nanostructures grown on copper substrate.

For investigation of the chemical purity of the synthesized product, elemental analysis of 3D CuO-ZnO hybrid structures was performed by energy dispersive X-ray spectroscopy (EDS). The obtained EDS spectrum reveals the composition of the Cu, Zn, and O elements without any other impurity elements, as shown in Figure 3. The strong peaks of the Cu, Zn, and O elements are exhibited in the EDS spectrum. The Cu element originated from the CuO substrate and the Zn was contributed by the flower-like ZnO hierarchical structures. Furthermore, the atomic ratios of Zn to O and Cu to O are both 1:1, respectively, and this observation proves that the stoichiometric ratio is maintained.

**Figure 4:**
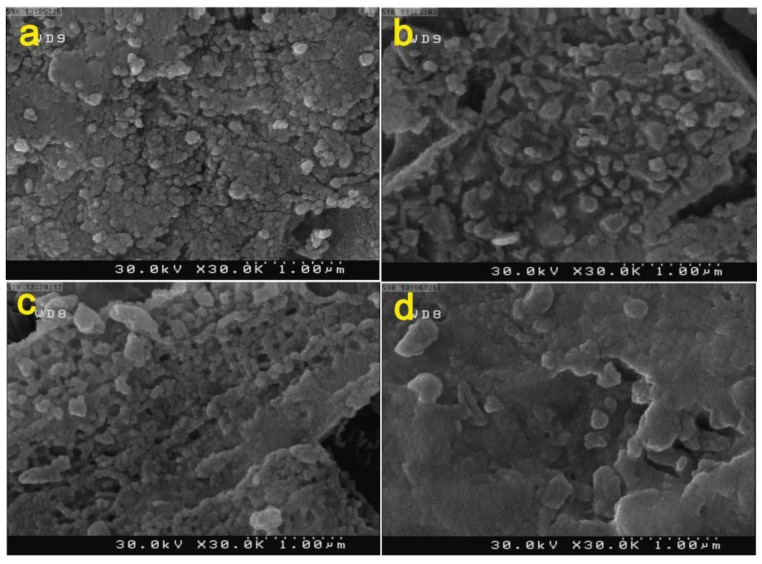
The SEM images of CuO nanostructures engraved on the Cu foil surface in different synthesis conditions. (**a**) Without any stabilizer or surfactants; and in the presence of (**b**) PEG 6000; (**c**) SDS; (**d**) CTAB.

Scanning Electron microscopy (SEM) was used to study the morphology of the products. In the SEM technique, a focused electron beam scans the conductive sample surface and reveals information about the sample including the external morphology (texture) and topography. Figure 4 shows the SEM images of the CuO nanostructures engraved on the Cu foil surface in different synthesis conditions. It is clearly observed from the SEM images that the copper foils were covered with films consisting of a large number of nanoparticles. Figure 4a indicates that the C0 sample synthesized from chemical oxidation has a sphere like structure. According to Figure 4a, the nanoscale roughness of the copper surface can clearly be seen and indicates that after interaction with the EG agent, the copper surface is covered with numerous nanoparticles which are typically 50–90 nm in size.

As shown in Figure 4b, when PEG 6000 was used as capping agent (C1 sample), a combination of coalesced particles and bulk structures are made. We propose that because PEG 6000 is a macromolecule with a high molecular weight, it acts as an obstacle by hindering the contact between the clean Cu foil surface and the EG solvent. Irregular distributions of the concentration of PEG 6000 form a disordered pattern of CuO structures on the Cu foil substrate. For the sample with SDS as the anionic surfactant (C2 sample), the SEM image shows that sponge-like CuO has been obtained (Figure 4c). It seems that after formation of the primary nucleus, this surfactant is adsorbed preferentially on the nuclei surface, and inhibits the accessibility of the surface for reactants by the steric hindrance mechanism [21]. Additionally, agglomerated and impacted structures were obtained by using CTAB as a cationic surfactant as shown in Figure 4d (sample C3). The formation of dense structures in Figure 4d using CTAB is associated with its cationic head group, and this surfactant easily interacts with free oxygen groups on the surface of the CuO substrate and increases the agglomeration of structures [22]. The SEM analysis indicates that the used surfactants operate as preventing agents for ethylene glycol to adhere to the Cu substrate. Therefore, using surfactants with high molecular weight and those which have strong interactions with the substrate is not recommended.

### 3.1. The Mechanism of CuO Formation

**Figure 5:**
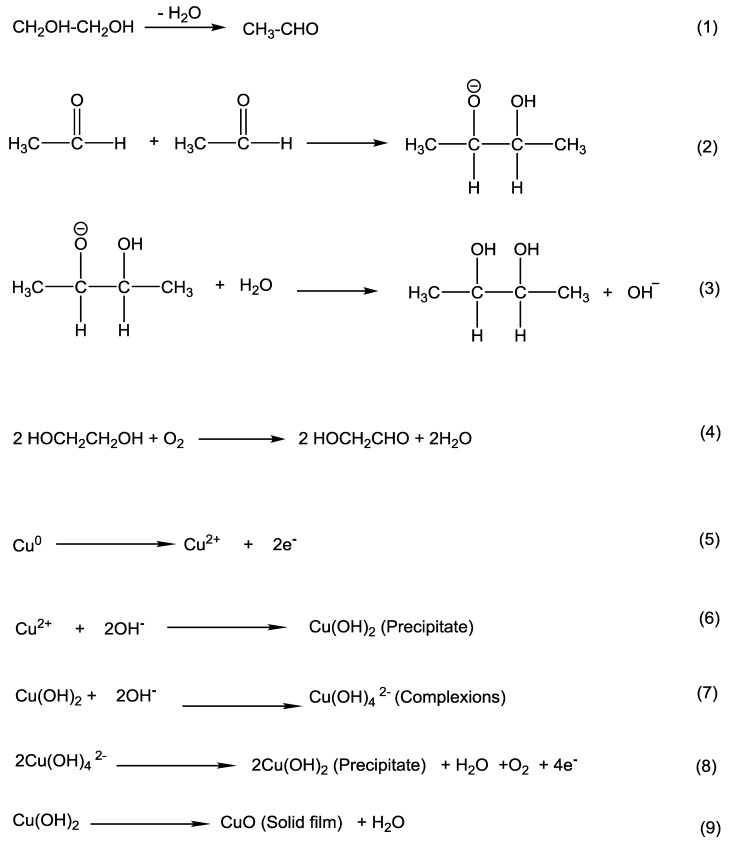
The proposed mechanism of CuO formation.

The compound for starting the synthesis of CuO is hydroxide. The main feature of the reaction mechanism is that the reaction proceeds via the solution phase rather than the solid phase, and the metal particles are formed by nucleation and growth from the solution. As described in Figure 5 (Equations (1)–(3)), the formation of the main product, diacetyl, can be explained in terms of a double oxidation of acetaldehyde, previously produced by the dehydration of ethylene glycol [23].

In addition, the produced OH^−^ ions can react with metal ions for the formation of metal hydroxide (Equation (4)) [23,24]. Generally, the proposed reactions for the growth of CuO nanoparticles essentially consist of two stages of oxidation and dehydrogenation [25].

In the following, during the reaction, Cu foils as copper sources dissolved in the solutions and produced Cu^2+^ ions with two electrons left behind. Previous investigations have proven that the oxidation rate occurs quickly, and the Cu^2+^ ions are continuously released into the solution [26]. During the growth in a solution of EG, the Cu foil was slowly reacted and oxidized to Cu(OH)_2_ in alkaline solution. It has been proven that Cu^2+^ ions prefer to arrange in a square-planar coordination with OH^−^. Therefore, the transition Cu(OH)_2_ product reacts with more hydroxyl groups to yield Cu(OH)_4_^2−^ species, which precipitate as Cu(OH)_2_ on top of the copper foils, and then decompose into CuO (solid structures) after dehydration at 80 °C for 5 days [27,28,29]. As is well-known, during the formation of CuO nanoarrays in solution, Cu(OH)_2_ usually serves as the precursor and template during the thermal dehydration process. Moreover, the Cu(OH)_2_ precipitate is a thermodynamically metastable phase, which can be easily transformed into the more stable CuO solid film. This transformation is found to take place in aqueous media at a relatively low temperature [20]. The mechanism reactions for the formation of CuO nanoarrays are as follows (Figure 5 Equations (5)–(9)):

### 3.2. Studying the Morphology and Growth Mechanism of the Flower Like ZnO Products

**Figure 6:**
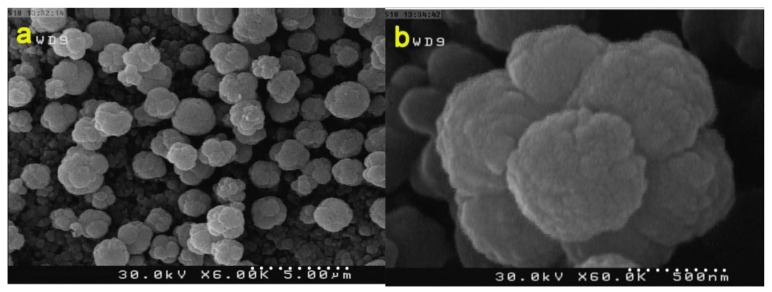
SEM images for the flower-like ZnO hierarchical nanostructures prepared without any surfactant or stabilizer at (**a**) 5 µm and (**b**) 500 nm magnifications.

Uniformly grown flower like ZnO hierarchical structures on the CuO substrate were confirmed using FE-SEM analysis. SEM images for the samples prepared without any surfactant or stabilizer (Z0 sample) at different magnifications are shown in Figure 6. The results indicate that flower like ZnO structures are partially grown on the CuO film (Figure 6a). The ZnO hybrid hierarchical structures are easily distinguishable from the CuO undercoat. The high magnification SEM image of the Z0 sample reveals that the flower-like ZnO has a special 3D structure that is stacked by seven mini-spheres. The average diameter of each petal of the ZnO microspheres is about 554 nm and the diameter of the total flower like structure is about 1.485 µm. Each mini-sphere contains numerous nanoparticles and the structures are symmetrical with specific shapes (Figure 6b).

**Figure 7:**
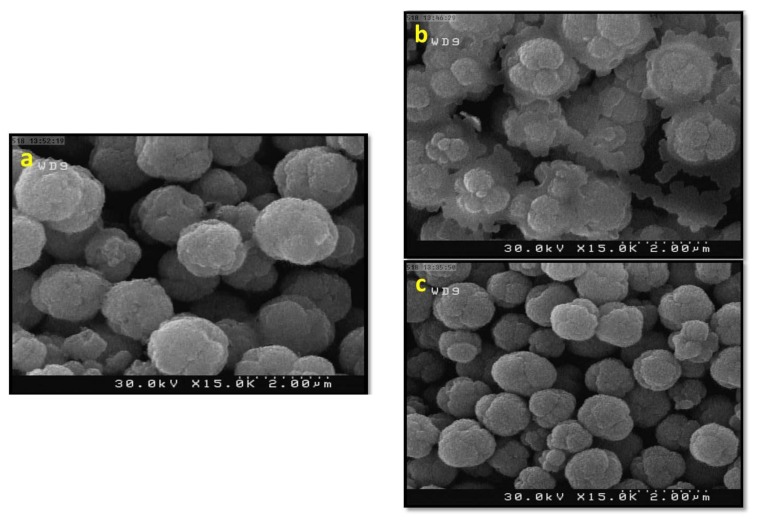
SEM images for the effects of surfactants and stabilizers on the final morphology of 3D CuO-ZnO hybrid hierarchical nanostructures. In the presence of (**a**) PEG 6000; (**b**) SDS and (**c**) CTAB.

Also, the effects of the surfactants and stabilizers on the final morphology of the 3D ZnO structures were studied. A representative SEM image of the 3D CuO-ZnO hierarchical structures is shown in Figure 7. According to Figure 7a, it is clear that when PEG 6000 is used as the capping agent (Z1 sample), the particles are aggregated and bulk structures are made. The flower like structure has also been destroyed and only micro spheres are obtained. In comparison with the Z0 sample, the microspheres are denser but the mean size of the spheres is approximately the same as the flower like structures (1.491 µm). 

The use of SDS as the surfactant had different impacts (Z2 sample). It seems that SDS leads to the formation of dome-shaped with flat base microstructures (Figure 7b). Some relics of the flower like symmetry still remain. Also in comparison with the Z0 sample, the mean size of the microstructures is increased (≈1.7 µm). The surface of the CuO nanospheres is almost completely covered by ZnO materials. The results prove that the existence of SDS has real effects on the diffusion and crystallization of the flower-like ZnO structures. Thus, it is apparent that sodium dodecyl sulfate can induce morphological changes in the ZnO aggregates in polar solvents [30]. Although the effect of SDS on the ZnO architecture is obvious, the origin of the interaction is more complex. Sodium dodecyl sulfate (SDS), is an amphiphilic molecule that has an anionic head group composed of a sulfate ion and a sodium counterion, while the surfactant tail is a linear hydrophobic alkyl chain [31]. We propose that the effect of SDS on the morphology of the ZnO aggregates is a result of the electrostatic shielding properties of the sulfate and sodium ions. Because of electrostatic repulsion, the anionic head group of SDS has a repulsive interaction with the –OH groups of the EG solvent. On the other hand, the long hydrophobic chain is not soluble in polar solvents such as EG. So SDS does not undergo self-assembly in the solvent mixtures used throughout this study, and interacts in a different way with the Zn^2+^ ions to precipitate on the CuO substrate.

**Figure 8:**
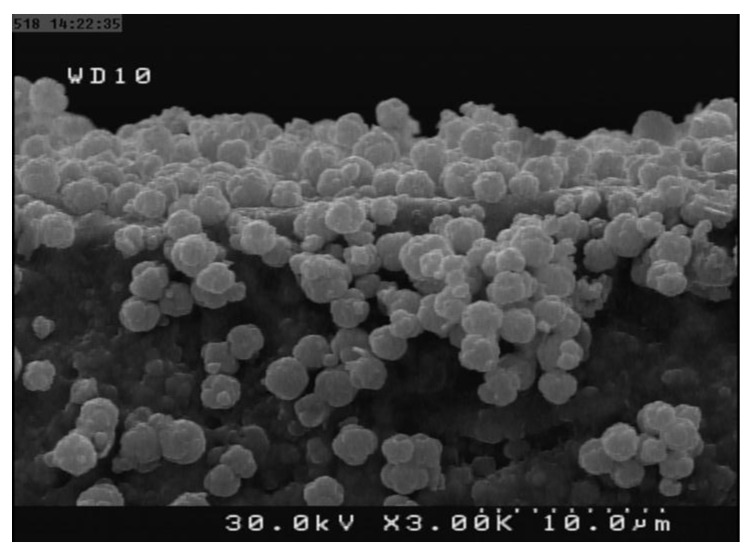
Cross section view of the flower-like CuO-ZnO hybrid hierarchical nanostructures prepared without any surfactant or stabilizer.

Figure 7c depicts the FE-SEM image of the sample Z3 synthesized using CTAB as the surfactant. As shown, the morphology of this sample is micro-particles with average sizes of about 1 µm. The morphology of the ZnO microstructures is changed and cabbage like structures are obtained. The formation of the small and dense structures is due to its cationic head group. CTAB easily interacts with the free oxygen groups on the surface of the formed ZnO nucleus and agglomeration and the final size of the products will decrease. On this basis, to obtain flower like ZnO architectures with better geometric symmetry, the use of these capping agents and surfactants are not recommended. Therefore, further investigations were continued with the Z0 sample. Figure 8 depicts the cross section view of the CuO-ZnO hierarchical structure. As is shown, a layer of flower like ZnO structures is uniformly covered on the CuO nanosphere substrate. The ZnO flowers have an average cross-section diameter and length of 1.8 µm and 1.9 µm, respectively. From the reaction process point of view, the reaction routes for the formation of flower like ZnO using Zn(acac)_2_ as a precursor are shown below (Equations (10)–(12)):

Zn^2+^ + 2OH^-^ ↔ Zn(OH)_2_ ↓ (10)

Zn(OH)_2_ + 4NH_3_ ↔ Zn(NH_3_)_4_^2+^ + 2OH^-^ (11)

Zn(OH)_2_ ↔ ZnO + H_2_O (12)

Basically, zinc acetate dissolves to produce Zn^2+^ ions. Obviously, at the beginning of the reaction, Zn(OH)_2_ precipitates were obtained (Equation (10)), evidenced by a white solid appearing in our experiment. After adding the NH_3_ solution, the Zn(OH)_2_ precipitate began to dissolve and a homogenous aqueous solution containing Zn(NH_3_)_4_^2+^ ions is obtained [32]. This solution is stable and transparent with a large amount of complexing agents. According to the equilibrium in Equation (2), after increasing the temperature, the total concentration of NH_3_ in the solution became lower and the reaction moves to the left to compensate [33]. Thereafter, Zn(OH)_2_ would transform into ZnO nuclei by the dehydration reaction under hydrothermal conditions (Equation (12)). Here, EG as the stabilizer agent prevented the amalgamation of the ZnO nuclei in the supersaturated solvents during the reaction process. Furthermore, EG can serve as the director for the growth of ZnO nanoparticles along certain direction, which leads to dispersed flower like structures with the appearance of white precipitation [34].

**Figure 9:**
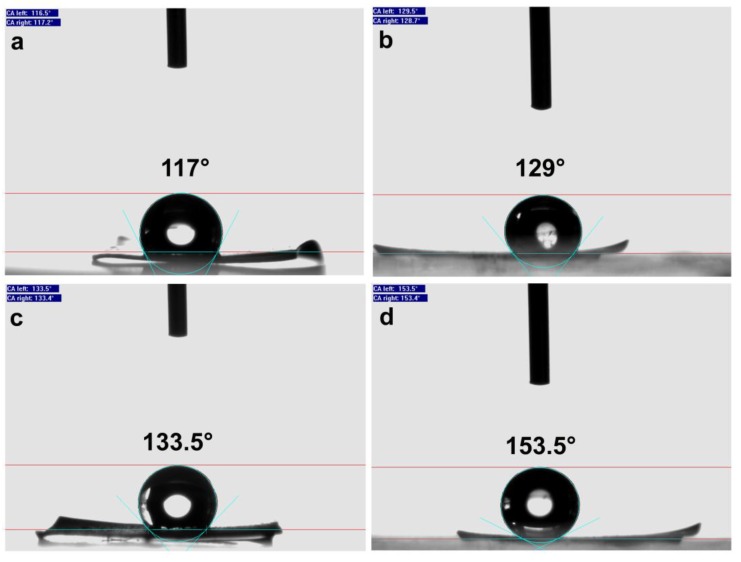
The shapes of a water droplet on different surfaces and the corresponding contact angle (CA) values: (**a**) modified CuO nanospheres surface; (**b**) modified underdeveloped CuO-ZnO micro-flower surfaces; (**c**) modified flat CuO-ZnO surface by SA; (**d**) modified flat CuO-ZnO surface by PDMS.

It is commonly confirmed that the unusual wetting properties of superhydrophobic surfaces are dependent on both their chemical composition and geometric surface microstructures [35]. In addition, the characteristic of wettability of a surface can be well-controlled through the combination of surface roughness and different chemical modifications. It is expected that as-prepared flower-like ZnO-CuO architectures with special micro-nanostructures may result in a particular wettability. Considering the micro and nanoscale binary hierarchical architecture of the surfaces of CuO-ZnO covered Cu foil, the wetting properties of the CuO-ZnO microstructures obtained on the Cu foil during reactions were evaluated using contact angle (CA) measurements. Figure 9 shows the captured micrographs of a water droplet on the surface of the CuO-ZnO films. As can be seen in Figure 9a, for the CuO film, the corresponding CA is determined to be 117°, indicative of the surface hydrophobicity. 

The water CA was found to increase from 117° for the nanostructured CuO film to 129° for the flower like CuO-ZnO microstructures (Figure 9b), indicative of the good hydrophobicity of the CuO-ZnO surface. Previously, our SEM results also confirmed that the ZnO microflowers with higher surface roughness demonstrate better hydrophobicity. This observation confirms that the persistence of the geometric structures of the ZnO micro flowers after the oxidation of the copper foil is essential for improving hydrophobicity. It is well known that for a specific surface, CA is strongly associated with both the surface roughness and surface energy, and the main factors that controls the surface energy is usually the type and properties of the surface functional groups [36]. For increasing the contact angle of the water droplet, the surface of the as prepared flower like ZnO hierarchical structures on the CuO substrate was further modified as follows; an as prepared CuO-ZnO foil was also modified with a stearic acid (SA) layer. Therefore, the as prepared CuO-ZnO substrate was immersed in 10 mL of 0.05 M SA ethanol solution for 1 h at room temperature. Finally the modified substrate was dried at 70 °C for 2 h and stored at room temperature. The result of the CA measurement is depicted in Figure 9c. As seen, the combination of the low surface energy SA and surface roughness (arising from the CuO nanoparticles and flower like ZnO micro structures) leads to obtaining a superhydrophobic surface with the static water contact angle of approximately 133.5°. In the other reaction, the surface of the as prepared CuO-ZnO substrate was modified with polydimethylsiloxane (PDMS). The intended substrate was put in a 3% (*v*/*v*) solution of PDMS in toluene for 1 h at room temperature. The foil was dried at 70 °C for 2 h and stored at room temperature and then its wettability was investigated. The results for the CA measurement confirmed that the as modified substrate showed excellent superhydrophobicity with a water contact angle as high as 153.5° (Figure 9d). For this sample, the water droplets can roll off the surface very quickly when the substrate is slightly crooked. Moreover, according to the previous studies, it should be noted that the rough surfaces without modification usually do not have a superhydrophobic property and this suggests that a suitable micro- nanoscale binary surface structure only is not enough to achieve a superhydrophobic surface [35].

**Figure 10:**
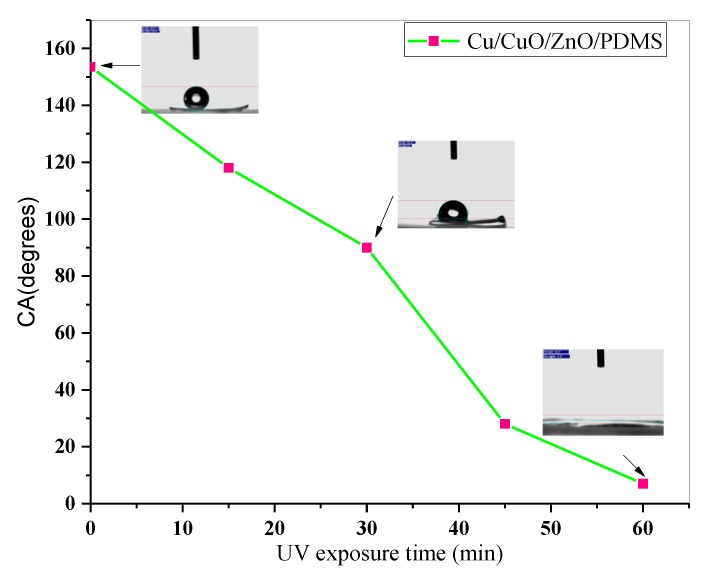
Water contact angle as a function of the UV irradiation time for the Cu/CuO/ZnO/PDMS sample.

One of the most advantageous characteristics of the superhydrophobic film was its tunable wettability when it is exposed to UV irradiation. Here, in order to study the UV-enhanced wettability conversion of the CuO/ZnO/PDMS coating, the sample (surface area of 1 cm^2^) was placed under a UV light source (400 W mercury lamp) with a working distance of 10 cm in ambient conditions. The photodecomposition process and water contact angles as a function of the UV illumination time are shown in Figure 10. As shown, after exposing the sample to UV light for 30 min, the contact angle was decreased to about 4° and the sample nature was switched from superhydrophobic to superhydrophilic. However, when the UV-irradiated sample had been dried in a dark place at 80 °C for 24 h, the water contact angle (WCA) was increased again and the sample recovered its pristine superhydrophobic state with a contact angle of about 150°.

**Figure 11:**
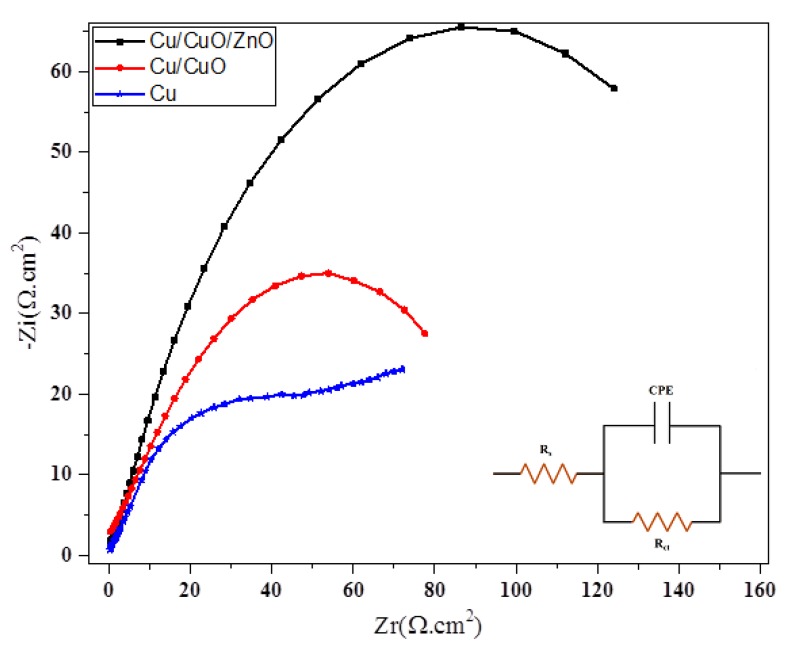
Nyquist plot for the bare Cu foil, Cu/CuO, and Cu/CuO/ZnO electrodes in 3.5% NaCl solution. The Cu/CuO/ZnO sample showed a better anticorrosion property.

**Figure 12:**
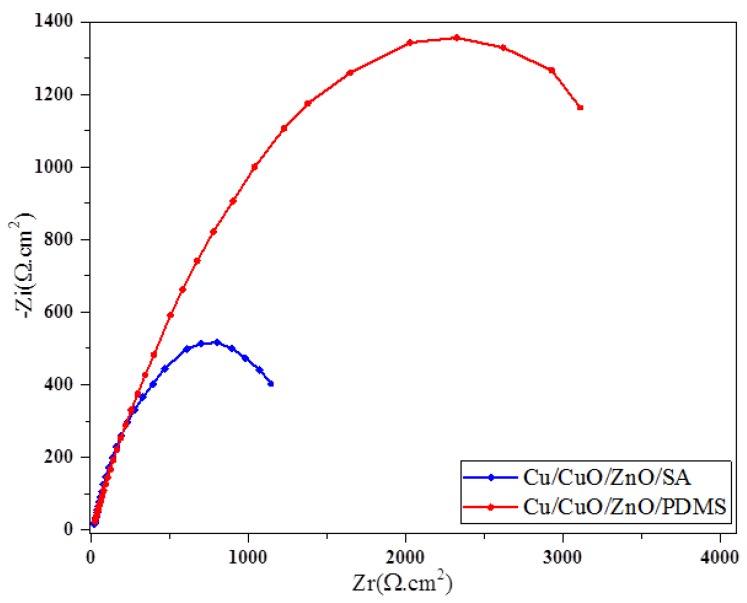
Nyquist plot for the modified Cu/CuO/ZnO/SA and Cu/CuO/ZnO/PDMS electrodes in 3.5% NaCl solution. The CuO/ZnO/PDMS sample showed a higher anticorrosion efficiency compared to the other samples.

For investigation of the corrosion resistance of the resulting surfaces, EIS was used as a nondestructive and useful technique to characterize the electrochemical reactions that occurred at the metal/salt solution boundary. Figure 11 shows the EIS results and typical Nyquist impedance plots of the bare copper and surface-treated copper oxide after 60 min of exposure in a 3.5 wt % aqueous NaCl solution. As we know, the low frequency impedance is known as the Warburg impedance (W). This impedance shows that the dissolution mechanism of copper is controlled by the mass transport rate. The diffusion step of copper dissolution in several papers is ascribed to the transport of the transportation of soluble cuprous chloride complexes from the surface of copper to the bulk solution. Moreover the depressed capacitive loop has been ascribed to roughness and in-homogeneities on the surface during corrosion. Figure 11 also shows that the Warburg impedance disappears at low frequencies when the surface of the bare copper is oxidized to CuO or when flower like ZnO structures are grafted on the surface. The Nyquist diagrams show a semicircle in the high frequency range related to the resistance of charge transfer (Rct) followed by a straight line in the low frequency region related to the double layer capacitance (C). As seen, the diameters of the Nyquist loop of the flower like ZnO structures on the Cu is significantly larger in compare with the bare copper and Cu/CuO sample. This observation demonstrates that the copper corrosion is controlled by the charge transfer process as the CuO/ZnO layer is added. The EIS results for further surface modification with SA and PDMS are shown in Figure 12. It is observed that the diameter of the semicircle Nyquist impedance plots increases obviously in the presence of the SA and PDMS as inhibitors, which increases the corrosion resistance of the sample. Obviously, in Table 2, the R_ct_ values increase with the modification of the surface. Consequently, the inhibition efficiencies (*η*) of the CuO, CuO/ZnO, CuO/ZnO/SA, and CuO/ZnO/PDMS calculated from Equation (1), are around 44.61, 87.49, 97.47, and 99.79%, respectively, after the initial 60 min of exposure. These results indicate that the superhydrophobic CuO/ZnO/PDMS surface has dramatically enhanced the corrosion resistance of the copper foil.

## 4. Conclusions

In this work, CuO-ZnO hierarchical nanostructures on copper substrates have been prepared by the oxidation of copper foil in EG solutions at a moderate temperature of 100 °C. The flowerlike ZnO microstructures are formed on copper oxide foils easily via a glycothermal method. The results indicate that EG acts as both an alkaline agent and reductant for the growth of CuO nanostructures and unusual flower like ZnO microstructures. The XRD pattern of the product grown on the upward surface of the copper foil confirms the true synthesis of the monoclinic structure for CuO and the hexagonal structure for flower-like ZnO. The SEM image results confirmed that the used surfactants (SDS, PEG 600, and CTAB) operate as an obstacle for ethylene glycol and prevent to reaction with the Cu substrate. Therefore, using surfactants with high molecular weight and those which have a strong interaction with the substrate is not recommended for the synthesis of both CuO and ZnO in the mentioned conditions. The surface hydrophobicity/superhydrophobicity was achieved on the modified Cu foils because of the combination of the uniform surface nano or microstructure and low surface energy. The results for the CA measurements confirmed that modifying the obtained rough surfaces of CuO and Cu-ZnO promotes the hydrophobic property, and using just a 3% solution (*v*/*v*) of PDMS in toluene leads to obtaining excellent superhydrophobicity with a water contact angle as high as 153.5°. Due to the high hydrophobicity of the surface, the obtained anticorrosion efficiency for the CuO/ZnO/PDMS sample was about 99%. These results provide valuable information for the design of the patterned superhydrophobic surfaces through a simple approach and multifunctional materials, which have many potential applications in corrosion protection, sensors, energy storage devices, and self-cleaning and anti-icing coatings.

## Figures and Tables

**Table 1:** Preparation conditions for all of the samples.

Sample	Stabilizer or Surfactant	Temperature (°C)	Time (h)	Morphology
C0	-	80	120	Rough surface with nanoscale particles
C1	PEG 6000	80	120	Coalesced particles and bulk structures
C2	SDS	80	120	Sponge-like
C3	CTAB	80	120	Agglomerated and impacted structures
Z0	-	100	8	Symmetry flower-like microstructures
Z1	PEG 6000	100	8	Symmetry cabbage-like microstructures
Z2	SDS	100	8	Asymmetry microstructures
Z3	CTAB	100	8	Non-sized cabbage-like microstructures

**Table 2:** Impedance parameters and anticorrosion efficiencies of different samples in 3.5% NaCl solution.

Sample	R_s_ (Ω·cm^2^)	R_ct_ (Ω·cm^2^)	C (μF·cm^2^)	η%
Cu	14 ± 4%	81.3 ± 4%	79.2 ± 6%	-
Cu/CuO	16.62 ± 4%	146.8 ± 4%	69.7 ± 7%	44.61
Cu/CuO/ZnO	23.58 ± 10%	650 ± 5%	27.4 ± 7%	87.49
Cu/CuO/ZnO/SA	35.9 ± 9%	3220 ± 10%	7.06 ± 8%	97.47
Cu/CuO/ZnO/PDMS	651 ± 8%	40,500 ± 9%	3.61 ± 7%	99.79
